# Supplementary material for: Management of veterinary anaesthesia and analgesia in small animals: A survey of English-speaking practitioners in Canada
Source: PLoS One. 2021 Sep 28;16(9):e0257448. doi: 10.1371/journal.pone.0257448 (PMC8478190; doi:10.1371/journal.pone.0257448)
Supplement: S1 Appendix — Presentation of the questionnaire used for the electronic survey, with the different sections, and all questions. (DOCX) [file pone.0257448.s001.docx]

**S1 – APPENDIX: QUESTIONNAIRE**

**PART I - TYPE OF PRACTICE**

Question 1: You are:

- Male
- Female

Question 2: When did you obtain your veterinary degree (DVM)?

- <15 years
- >15 years

Question 3: You practice:

- Alone
- In a veterinary clinic with 2 – 4 veterinarians
- In a veterinary clinic with more than 4 veterinarians

Question 4: What is your on-call schedule?

- Never
- 1 week or 1 day out of 3
- 2 weeks or 2 days out of 3
- 100 % of the time
- Other

Question 5: Do you practice in:

- A very big city (>100 000 habitants)
- A large city (50 001-100 000 habitants)
- A city (10 000-50 000 habitants)
- A small town or village (<10 000 habitants)

Question 6: You practice in:

- A first line-clinic
- A referral center

Question 7: On average, on a daily basis, how many patients are anesthetized in your practice?

- 0-1
- 2-3
- 4-6
- 7-9
- 10 +

**PART II - EVALUATION AND MANAGEMENT OF ANAESTHETIC RISK**

Question 8: Do you provide handouts or other supporting material with the explanation of the importance of anaesthesia and related risks to the animal?

- Yes
- No

Question 9: If yes, who explains potential risks of anaesthesia to the pet owner?

- Secretary
- Animal Health Technician
- Veterinarian

Question 10: Indicate your agreement with the following statement: **Animals rarely need analgesia during recovery**.

- Yes
- No

Question 11: Do you give the choice to the client for postoperative pain management?

- Yes
- No

Question 12: If yes, who gives the choice to the client for postoperative pain management?

- Secretary
- Animal Health Technician
- Veterinarian

Question 13: Is an owner’s written consent obtained before surgery?

- Yes
- No

Question 14: What is the percentage of owners expressing their concern about anaesthesia?

- Less than 10%
- 10-40%
- 41-60%
- 61-80%
- More than 80 %

Question 15: Solid diet – How do you perform pre-anaesthetic fasting: No fasting or 6-12h before anaesthesia or 4h (or less) before anaesthesia. (Please check all that apply)

- All patients
- Healthy
- Paediatrics
- Debiliated/geriatric
- Other (please specify)

Question 16: Water - How do you perform pre-anaesthetic fasting: No fasting or 6-12h before anaesthesia or 4h (or less) before anaesthesia. (Please check all that apply)

- All patients
- Healthy
- Paediatrics
- Debiliated/geriatric
- Other (please specify)

Question 17: For what kind of patients would you perform a physical examination? (Please check all that apply)

- Any animal to be anaesthetized
- Young patients
- Old patients
- Debilitated patients

Question 18: Please indicate what parameters you monitor (or evaluate) in your physical examination. (Please check all that apply)

- All parameters
- Heart rate
- Heart auscultation
- Chest auscultation
- Peripheral pulse rate concomitant to heart auscultation
- Respiratory rate
- Lymphatic nodes palpation
- Abdominal palpation
- Temperature monitoring (rectal or other)
- History (systematically including appetite, water intake, feces and urine)

Question 19: How long before anaesthesia do you perform physical examination? Less than 24h or 1-7 days before or more than 7 days before:

- Routine surgery
- Non-elective surgery

Question 20: For what kind of patients would you recommend additional diagnostic work-up or laboratory profile? (Please check all that apply)

- Any patient to be anaesthetized
- Young patients
- Old patients
- Debilitated patients
- When I think it is necessary

Question 21: What percentage (0-20 %, 21-40 %, 41-60 %, 61-80 %, 81-100 %) of your clients would agree with your recommended additional diagnostic and/or laboratory exams?

- For a patient you believe in good health
- For a young patient
- For a geriatric patient
- For a patient you believe at risk

Question 22: Which of the following diagnostic and/or laboratory exams would you recommend for healthy patient, paediatric patient, geriatric patient and patient you believe at risk? (Please check all that apply)

- None
- Haematocrit/total protein ‘’in-house’’
- Basic haematology (CBC)
- Serum biochemistry – liver enzymes
- Serum biochemistry – urea / creatinine
- Biochemistry – glycaemia
- Urine analysis
- ECG
- X-rays
- Electrolytes
- Other (please specify)

Question 23: Do you evaluate (Yes or No) ASA (American Society of Anesthesiologists) risk for...

- Routine surgery?
- Non-elective surgery?

Question 24: Do you prepare in advance emergency drugs?

- Yes, all of the time
- Only for procedures you consider at-risk
- Never

Question 25: Do you have a box/kit with emergency drugs and supplies for cardiopulmonary resuscitation?

- Yes
- No

Question 26: Among the following drugs, which one (Yes or No) do you use? (Please check all that apply)

- Adrenalin (epinephrine)
- Atropine
- Phenylephrine
- Ephedrine
- Dobutamine
- Dopamine
- Doxapram
- Glycopyrrolate
- Vasopressin

If Yes, how often? Once a year or less or once every 6 months or once a month or once a week or more

- Adrenalin (epinephrine)
- Atropine
- Phenylephrine
- Ephedrine
- Dobutamine
- Dopamine
- Doxapram
- Glycopyrrolate
- Vasopressin
- Other (please specify)

Question 27: Which of the following antagonist agents (Yes or No) do you currently have available? Only select those you are certain to possess. If you do not use drugs that can be reversed, please check "N/A" when applicable.

- Naloxone
- Atipamezole
- Yohimbine
- Tolazoline
- Flumazenil

**PART III - ANAESTHESIA PROCEDURE**

Question 28: How do you generally plan your premedication?

- Specific preparation for each single anaesthesia, doses vary among patients
- Same protocol for all patients and drugs mixed just before administration
- Premix (use of a drug combination that are prepared beforehand with known concentrations and volumes/kg)
- I do not use any premedication

Question 29: Which of the following drugs are part of your premedication? (Please check ally that apply). Please do not check unless certain to possess the selected drug. Frequency (%) of use in routine surgeries (0 %, 1-20 %, 21-40 %, 41-60, 61-80 %, 81-99 %, 100 %).

- Acepromazine (Atravat^®^, Acevet^TM^, etc.)
- Diazepam (Valium^TM^)(if available)
- Midazolam (Versed^®^)(if available)
- Glycopyrrolate (if available) / atropine
- Dexmedetomidine (Dexdomitor^TM^)
- Metetomidine (Domitor^®^, Cepetor^TM^)
- Xylazine (Rompun^®^, AnaSed^®^, etc.)
- Morphine (if available)
- Hydromorphone (if available)
- Butorphanol
- Buprenorphine
- Fentanyl (patch or injectable)
- NSAID: Meloxicam, carprofen, deracoxib, firocoxib, robenacoxib, ketoprofen, tolfenamic acid, etc.)

Used (Yes or No) for non-elective surgery:

- Acepromazine (Atravat^®^, Acevet^TM^, etc.)
- Diazepam (Valium^TM^)(if available)
- Midazolam (Versed^®^)(if available)
- Glycopyrrolate (if available) / atropine
- Dexmedetomidine (Dexdomitor^TM^)
- Metetomidine (Domitor^®^, Cepetor^TM^)
- Xylazine (Rompun^®^, AnaSed^®^, etc.)
- Morphine (if available)
- Hydromorphone (if available)
- Butorphanol
- Buprenorphine
- Fentanyl (patch ou injectable)
- NSAID : Meloxicam, carprofen, deracoxib, firocoxib, robenacoxib, ketoprofen, tolfenamic acid, etc.)
- Other (please specify)

Question 30: Which of the following induction agent(s) do you use? (Please check all that apply). Please do not check unless certain to possess the selected drug. Frequency (%) of use in routine surgeries (0 %, 1-20 %, 21-40 %, 41-60, 61-80 %, 81-99 %, 100 %).

- Alfaxalone (Alfaxan^®^)
- Ketamine (Ketaset^®^, VetalarTM, Ketalean^®^, etc.) used alone
- Ketamine -diazepam(Valium^TM^)
- Ketamine-(dex)medetomidine
- Propofol (Rapinovet^®^, etc.)
- Thiopental (Thiotal^®^, etc.)

Used (Yes or No) for non-elective surgery:

- Alfaxalone (Alfaxan^®^)
- Ketamine (Ketaset^®^, Vetalar^TM^, Ketalean^®^, etc.) seule
- Ketamine-diazepam (Valium^TM^)
- Ketamine-(dex)medetomidine
- Propofol (Rapinovet^®^, etc.)
- Thiopental (Thiotal^®^, etc.)
- Other (please specify)

Question 31: Do you use injectable anaesthesia for maintenance of anaesthesia?

- Yes
- No

Question 32: If yes, which of the following drugs are you using? (Please check all that apply) Do not check unless certain to possess the drug.

- Alfaxalone (Alfaxan®)
- Ketamine (Ketaset®, Vetalar™, Ketalean®, etc.)
- Propofol (Rapinovet®, etc.)
- Other (please specify)

Question 33: For which procedure and for what kind of patient do you use an injectable-only anaesthesia? Please explain. - Open-Ended Response

Question 34: What kind of inhalant anaesthetic do you use?

- Isoflurane
- Sevoflurane
- I do not use inhalant anaesthetic
- Other (please specify)

Question 35: What type of anaesthesia machine is available in your practice (Please check any that applies)?

- Bain circuit (also called: semi-open or non-rebreathing)
- Circle breathing circuit (also called: semi-closed or rebreathing or with CO2 absorber)
- None
- Other (please specify)

Question 36: At what point do you administer an NSAID for routine surgery?

- Never
- At the same time you give premedication
- At the same time you give premedication
- During surgery, before incision
- During surgery, after incision
- During recovery

Question 37: Do you use constant rate infusion of analgesic agent?

- Yes
- No

Question 38: If yes, which agent? (Please check all that apply)

- Fentanyl
- Ketamine
- Lidocaine
- Other (please specify)

Question 39: During routine surgery, do you perform loco-regional anaesthesia?

- Yes
- No

Question 40: If yes, please specify which block is commonly performed (please check all that apply).

- Maxillary block
- Mandibular block
- Infra-orbital block
- Mental block
- Ring block for declawing
- Other (please specify)

Question 41: How do you use NSAID?

- Single injection
- Over 3-4 days
- Over 7 days
- Never

Question 42: How do you use opioid?

- Single injection
- As needed
- Always one injection during recovery, then as needed
- Never

Question 43: Do you use opioid and NSAID together?

- Yes
- No

Question 44: If you use NSAID, please specify which one is used most often for post-operative analgesia (cat and dog):

- Meloxicam
- Carprofen
- Deracoxib
- Firocoxib
- Robenacoxib
- Ketoprofen
- Tolfenamic acid
- Other (please specify)

Question 45: If you use opioids, please specify which one is used most often for post-operative analgesia (cat and dog):

- Hydromorphone
- Morphine
- Buprenorphine
- Butorphanol
- Other (please specify)

**PART IV - MONITORING**

Question 46: During general anaesthesia in dogs and cats. How often (0 %, 1-20 %, 21-40 %, 41-60, 61-80 %, 81-99 %, 100 %) do you perform:

- Intravenous catheterization
- Endotracheal intubation
- Fluidtherapy
- Pre-oxygenation
- Use of a fluid pump

Question 47: During injectable anaesthesia. Do you provide oxygen to the patient?

- Yes, by putting the oxygen supply under the patient’s nose
- Yes, by using a mask
- Yes, after intubation, the patient is then connected to an anesthesia machine
- No

Question 48: How do you monitor cardiovascular function during general anaesthesia? (Please check all that apply) - Heart rate (calculated during auscultation or measured by an equipment)

- Heart rate (calculated during auscultation or measured by an equipment)
- Peripheral arterial pulse
- ECG
- Cardiac auscultation
- Non-invasive blood pressure measurement (oscillometric device (e.g. PetMAP™) or Doppler)
- Capillary refill time / mucous membranes colour
- Other (please specify)

Question 49: How do you monitor respiratory function during general anaesthesia? (Please check all that apply)

- Respiratory rate (visualisation of thoracic movement / ventilation bag of the anaesthesia machine)
- Pulmonary auscultation
- Respiratory rate measured with a monitor
- Pulse oximeter (SpO2 (haemoglobin oxygen saturation)
- Capnography / capnometry (End-Tidal CO2)
- Other (please specify)

Question 50: Out of the above-mentioned cardiovascular or respiratory parameters, which would you evaluate first to detect a ventilatory or peripheral perfusion issue? Please, explain (Indicate what comes naturally to your mind) - Open-Ended Response

Question 51: How do you monitor neurological function during general anaesthesia? (please check all that apply)

- Jaw tone
- Ocular reflex, palpebral reflexes
- Eye position
- Pharyngeal reflex (swallowing)
- Withdrawal reflex

Question 52: What is the current status (Present at the clinic or Present and used in routine cases or Present and used in non-elective cases) of the following equipment monitors in your clinic? (please check all that apply)

- Pulse oximeter (SpO2 and heart rate)
- ECG
- Oesophageal stethoscope
- Apnea monitor (ApAlert™)
- Capnograph/ Capnometer (ETCO2)
- Blood gases analyzer
- Oscillometric blood pressure measurement (e.g., PetMAP™ )
- Doppler blood pressure measurement
- Invasive blood pressure measurements
- Multi-parametric monitor
- Other (please specify)

Question 53: If complications arise during general anaesthesia, do you have means to perform additional diagnostic and laboratory exams "in house" (staff and equipment readily available)?

- Yes
- No

Question 54: If yes, which of the following (Please check all that apply):

- All these exams
- Haematocrit / total protein
- Haematology
- Biochemistry
- Glycaemia
- Blood gases analysis
- Electrolytes measurement
- Blood typing
- Cross match for transfusion

Question 55: If needed, can you perform transfusion on a patient during anaesthesia (material rapidly available)?

- Yes
- No

Question 56: Does your clinic have a mechanical ventilator?

- Yes
- No

Question 57: If yes, in which of the following situations are you using it? (Please check any that apply)

- Routinely (over 50% of patient)
- In case of respiratory arrest during anaesthesia
- In case of patient is difficult to ventilate
- Never

Question 58: In your practice, who is in charge of anaesthetic monitoring for Routine surgery or Non-elective surgery?

- Dedicated staff
- The same person who helps during surgery
- The person performing surgical procedure

Question 59: In your practice, at which frequency is anaesthetic monitoring performed?

- Every 5 minutes
- Every 10 minutes
- Every 15 minutes
- At the beginning and the end of anaesthesia
- No fixed frequency, monitoring is done as anaesthesia goes

Question 60: Do you routinely use an anaesthetic record?

- No
- Yes, systematically

If yes, what are the recorded vital signs?

Question 61: What do you monitor during anaesthetic recovery? (Please check all that apply)

- Body temperature
- Visual parameters (eye position, mucous membranes colour, thoracic movements, etc.)
- Palpation (pulse quality, jaw tone, palpebral reflex, etc.)
- Auscultation (heart rate and rhythm, respiratory rate)
- Same monitoring as during anaesthesia

Question 62: For how long do you perform such monitoring? (Please check all that apply)

- Until extubation
- Until temperature increases to a level you consider as normal
- Until prompt ability to hold its head in sternal position

Question 63: When do you extubate the patient?

- Right after the end of anaesthesia
- As soon as the animal is transferred to the recovery room
- When gag reflex is patent
- When the animal chews the endotracheal tube

Question 64: For routine surgery, what is your cut-off temperature in order to stop warming up a patient?

- 36°C
- 37°C
- 38°C
- We don’t always monitor body temperature

Question 65: Which of the following is available for warming up of patients?

- Forced air warmer (for instance, Bair Hugger)
- Hot water heating mats
- Electric plates / mats
- Heating lamp
- Fluid heater
- I do not warm up patients
- Other (please specify)

Question 66: How long do you usually keep your patient hospitalized following anaesthesia after routine surgery?

- Less than 6h
- 6 – 12h
- 12 – 24h
- More than 24h
