# Supplementary material for: Management of veterinary anaesthesia and analgesia in small animals: A survey of English-speaking practitioners in Canada
Source: PLoS One. 2021 Sep 28;16(9):e0257448. doi: 10.1371/journal.pone.0257448 (PMC8478190; doi:10.1371/journal.pone.0257448)
Supplement: Attachment — Submitted filename: PLOS one review.docx [file pone.0257448.s005.docx]

Overall, I think this information is extremely useful for anesthesiologists to help guide our teaching practices and where to emphasize specific aspects of anesthesia within the curriculum. It is very eye-opening and important to our current knowledge base about veterinary anesthesia. Aside from the more specific comments below, I have a couple general comments to consider. One is I think I would consider altering the conclusion a bit as I don’t think your numbers truly represent the population of veterinarians. Perhaps consider stating a sig. proportion of those sampled, I think that would be more appropriate. In addition, the manuscript seems very lengthy. Perhaps consider using your tables more effectively to reduce the lengthy results section (e.g. state significance and p values in tables rather than text). I also think there are several areas in the discussion that would be worth condensing in larger categories rather than individually discussing each aspect of the results. Also, there are several results restated in the discussion that could probably be removed.

Abstract

Line 32: Due to the low response rate I am not sure I would use the term significant. Perhaps just state a proportion of veterinarians surveyed. Also, I think need you need to specify what the other “class” of veterinarians you are referring to. For example, perhaps specify general practitioners or primary care veterinarians are the ones that do not follow current guidelines.

Introduction

Line 49 – Please specify “Canada” rather than this country.

Materials and methods

Line 76: Please describe more as to what dispomed did as a contributor for survey distribution. It is sort of ambiguous7 at this point.

Results

Line 102: I think it is important to consider that your demographic data may be skewed because there is no information describing the types of practice that the survey was distributed too via dispomed. Therefore, I don’t believe it is “interesting” that majority of respondents were GPs in large or small towns because that may have been the predominant facilities in which the survey was delivered too. Perhaps just state the findings of the demographics without drawing too much conclusions from it.

Table 1

Can you better define the term on-call hours? I think you mean 5pm-8a/weekend? Also, what would you define as episodic? Lastly, can you specify what you mean by “years of graduation”. I think you mean years since veterinary school graduation. Years of graduation doesn’t really define anything.

Line 135: can you define what was considered pediatric? Since geriatric is used often we should define this.

Line 158: Hindsight, but would have been interesting to identify the schools of graduation.

Line 161 – “blood” urea “nitrogen” evaluation

Table 3

What was classified or how was it determined for patients to be “believed at risk”?

“blood” urea “nitrogen and creatinine

Define the abbreviation ECG

Line 238 – this goes for all areas where only percentages are shown for comparison. Since the number of respondents that answered that specific question differ, do you think it would be of interest to your readers that you provide them with the exact count for comparison rather than or in addition to percent? I understand it gets to be a bit cumbersome, however im not sure in this situation percentage really tells us an entire lot. Just for example purposes, I think 3/10 is way different than 30/100 despite both being 30%.

Table 4- please provide number of respondents for each item.

Line 273: what was the most common opioid for those graduated >15 years ago? If there was a more common one.

Line 279 – infusion not perfusion

Line 282 “nerve” blocks

Line 283 “nerve” blocks

Line 284 – “infiltrative incisional line” and “ intratesticular””

Line 285 – please make sure we use appropriate terminology “nerve block”

Table 5 – what do you mean present in the clinic? Is that number of respondents? Please provide the numbers in all percentages. Again because it depends on the number of respondents I think it would be more useful. Also for the tables I think the title should be more descriptive. It really doesn’t say much about the study. In my opinion it should be stand alone so the reader can view the table and understand the basics of the study.

Results in general – I appreciate the work you put into providing the vast amount of detail in the results. It is fairly lengthy and I am afraid you will begin to lose some of the readers because of its length (maybe I am wrong). Perhaps with better descriptors in your tables you can shift some of this information including p values and statistical significance for comparison in tables and reduce the text length.

Discussion

Line 433 can we separate this sentence up into 2? Also specify younger graduates are more likely when compared to who? We assume older graduates but please specify.

Line 439 – I get what you are saying but I do not think I would write that in an open access journal based on it being a big speculation.

Line467 – I don’t agree with this statement. Yes, it does cause increased resistance but not if positive pressure ventilation is provided (by hand or machine). In addition the studies used to assess resistance are fairly old and used metal valves which were heavy. Our university does not use NRB circuits just because of the concern a student will accidently hit the quick flush.

Line 475 – I think simon et al 2017 on oligoanalgesia would be a good reference for this sentence.

Line 480-481 – I think you need to support this with a reference. Many areas routinely give them postoperatively so perhaps a brief explanation as to why this is inappropriate in canada.’

Line 490 – lets avoid restating the results in the discussion please.

Line 502 – inexpensive

line 508 – to administer “fluid”? be more specific

line 516- thanks for pointing this out

Line 521 – again too much results in the discussion. I understand there has to be some reference but in an already long manuscript I would try to reduce this when possible.

Line 535 – who’s recommendations?

line 602- please state a sig proportion of those surveyed… again I don’t think just over 100 vets is a sig portion of the population in Canada.
