# Supplementary material for: Management of veterinary anaesthesia and analgesia in small animals: A survey of English-speaking practitioners in Canada
Source: PLoS One. 2021 Sep 28;16(9):e0257448. doi: 10.1371/journal.pone.0257448 (PMC8478190; doi:10.1371/journal.pone.0257448)
Supplement: Attachment — Submitted filename: Response to reviewers_VF.docx [file pone.0257448.s006.docx]

**Response to the Reviewers’ comments on the manuscript**

**Title: Management of veterinary anaesthesia and analgesia in small animals: A survey of English-speaking practitioners in Canada**

**Ref:** PONE-D-21-05375

**Journal:** PLOS ONE

**Date:** 2021-05-17

______________________________________________________________________________

The authors would like to thank the Editorial Board and Reviewers for careful review of our manuscript and providing us with the comments to improve its quality. We have judiciously taken them into consideration in preparing our revision. The following detailed responses have been prepared to address the reviewer’s comments.

***Reviewer #1***

______________________________________________________________________________

**General comment:**

*Overall, I think this information is extremely useful for anesthesiologists to help guide our teaching practices and where to emphasize specific aspects of anesthesia within the curriculum. It is very eye-opening and important to our current knowledge base about veterinary anesthesia. Aside from the more specific comments below, I have a couple general comments to consider. One is I think I would consider altering the conclusion a bit as I don’t think your numbers truly represent the population of veterinarians. Perhaps consider stating a sig. proportion of those sampled, I think that would be more appropriate. In addition, the manuscript seems very lengthy. Perhaps consider using your tables more effectively to reduce the lengthy results section (e.g. state significance and p values in tables rather than text). I also think there are several areas in the discussion that would be worth condensing in larger categories rather than individually discussing each aspect of the results. Also, there are several results restated in the discussion that could probably be removed.*

**Response:**

The authors thank the Reviewer for his/her general comment and rigorous revision about the present manuscript and the recognition that the main subject is of interest. Regarding the conclusion, we appreciate Reviewer #1’s comment and agree that we should not automatically extrapolate our results to the entire English-speaking Canadian population. Therefore, we modified the conclusion accordingly, so it involves specifically the respondents and not the entire population.

We also made an effort to condense results section by avoiding repeating *P*-values and significance in both the tables and text. Several tables were added to present results as well. The discussion was revised and modified to decrease repetition of results.

Finally, we made many other changes following your comments for some areas of the manuscript, leading to a more concise and complete revised manuscript.

Minor point #1:

*Line 32: Due to the low response rate I am not sure I would use the term significant. Perhaps just state a proportion of veterinarians surveyed. Also, I think need you need to specify what the other “class” of veterinarians you are referring to. For example, perhaps specify general practitioners or primary care veterinarians are the ones that do not follow current guidelines.*

**Response:**

We thank the Reviewer #1 for this comment. We have made the corrections in the revised manuscript Lines 32-33.

Minor point #2:

*Line 49 – Please specify “Canada” rather than this country.*

**Response:**

Here, “this country” was replaced by “New Zealand” (Line 49) as this is the country studied in reference [8] Farnworth MJ, Adams NJ, Keown AJ, Waran NK, Stafford KJ. Veterinary provision of analgesia for domestic cats (Felis catus) undergoing gonadectomy: a comparison of samples from New Zealand, Australia and the United Kingdom. N Z Vet J. 2014;62(3):117–22. doi: 10.1080/00480169.2013.852447

Minor point #3:

*Line 76: Please describe more as to what Dispomed did as a contributor for survey distribution. It is sort of ambiguous at this point.*

**Response:**

Dispomed database was used to send the survey electronic link to all their small animal veterinarian customers. The information was specified in revised manuscript Line 76-77.

Minor point #4:

*Line 102: I think it is important to consider that your demographic data may be skewed because there is no information describing the types of practice that the survey was distributed too via dispomed. Therefore, I don’t believe it is “interesting” that majority of respondents were GPs in large or small towns because that may have been the predominant facilities in which the survey was delivered too. Perhaps just state the findings of the demographics without drawing too much conclusions from it.*

**Response:**

We thank Reviewer #1 for this comment and the sentence has been modified in the revised manuscript, Line 102-104.

Minor point #5:

*Table 1*

*Can you better define the term on-call hours? I think you mean 5pm-8a/weekend? Also, what would you define as episodic? Lastly, can you specify what you mean by “years of graduation”. I think you mean years since veterinary school graduation. Years of graduation doesn’t really define anything.*

**Response:**

“On-call hours” refers to moments when practitioners are not working at the clinic but can be called for a specific emergency and have to come in to assess patients or perform emergency surgery, whether during business hours or not. A complete definition was not provided in the questionnaire. “On-call hours” was changed for “on-call duty” and the definition was added in the manuscript.

“Episodic” was used to describe any other frequency than those already mentioned in the questionnaire. To avoid confusion, “Episodic” was replaced for “Other” and specific definitions were given in Table 1.

“Year of graduation” was indeed referring to “Years of practice since veterinary school graduation”. We thank Reviewer #1 to have point this out and we have made the correction in the revised manuscript in Table 1 and modified the legends (Line 107-112).

Minor point #6:

*Line 135: can you define what was considered pediatric? Since geriatric is used often we should define this.*

**Response:**
The provided definition was **“**Pediatric patients are classically under 3 months old, or as you define them in your practice”. No specific definition was provided in the questionnaire for “geriatric patients”, therefore it was indicated for the respondent “as you define them in your practice, for example 8 years for dog and 11 years old for cat”.

Minor point #7:

*Line 158: Hindsight but would have been interesting to identify the schools of graduation.*

**Response:**

Indeed, this would be an interesting risk factor to consider in future studies.

Minor point #8:

*Line 161 – “blood” urea “nitrogen” evaluation*

**Response:**

We thank Reviewer #1 for this comment, and we added this precision in the revised manuscript, Line 158 and Table 4.

Minor point #9:

*Table 3*

*What was classified or how was it determined for patients to be “believed at risk”?*

*“blood” urea “nitrogen and creatinine*

*Define the abbreviation ECG*

**Response:**

There was no definition provided in the questionnaire for “Believed at risk”. It was voluntarily left open and for the respondent to interpret. In retrospective, only 50% (57/115) respondent used ASA status, therefore a more precise definition might have yielded a lower response rate.

We added the precision to “Blood urea nitrogen” in Table 4 as suggested by Reviewer #1.

We replaced “ECG” for “Electrocardiogram” in Table 4 as suggested by Reviewer #1.

Minor point #10:

*Line 238 – this goes for all areas where only percentages are shown for comparison. Since the number of respondents that answered that specific question differ, do you think it would be of interest to your readers that you provide them with the exact count for comparison rather than or in addition to percent? I understand it gets to be a bit cumbersome, however im not sure in this situation percentage really tells us an entire lot. Just for example purposes, I think 3/10 is way different than 30/100 despite both being 30%.*

**Response:**

We thank Reviewer #1 for this comment. We agree with this point and therefore added all counts and number of respondents corresponding to percentages cited.

Minor point #11:

*Table 4- please provide number of respondents for each item.*

**Response:**

The number of respondents for each item was added in Table 4 (now Table 6 in revised manuscript) as recommended.

Minor point #12:

*Line 273: what was the most common opioid for those graduated >15 years ago? If there was a more common one.*

**Response:**

At Line 268-269 in Revised manuscript, hydromorphone is the most commonly used opioid for dogs by both respondents graduated over 15 years ago and less than 15 years ago, but a higher proportion of those graduated less than 15 years ago prefer it. The sentence was modified to clarify this information.

Minor point #13:

*Line 279 – infusion not perfusion*

**Response:**

The word “perfusion” was replaced by “infusion” as recommended at Line 276 in revised manuscript.

Minor point #14:

*Line 282 “nerve” blocks*

**Response:**

The word “nerve” was added at Line 280 in revised manuscript.

Minor point #15:

*Line 283 “nerve” blocks*

**Response:**

The word “nerve” was added at Line 281 in revised manuscript.

Minor point #16:

*Line 284 – “infiltrative incisional line” and “ intratesticular”*

**Response:**

We thank Reviewer #1 for this comment. The mentioned corrections were made at Line 281 in revised manuscript.

Minor point #17:

*Line 285 – please make sure we use appropriate terminology “nerve block”*

**Response:**

The word “nerve” was added at Line 283 in revised manuscript.

Minor point #18:

*Table 5 – what do you mean present in the clinic? Is that number of respondents? Please provide the numbers in all percentages. Again because it depends on the number of respondents I think it would be more useful. Also for the tables I think the title should be more descriptive. It really doesn’t say much about the study. In my opinion it should be stand alone so the reader can view the table and understand the basics of the study.*

**Response:**

“Present in the clinic” means the monitoring device is physically at the clinic and available for use by the practitioners, therefore this precision was added to the Table 8. Since the number or respondents was added for all percentages, the first column was deleted. The title of all tables were adjusted accordingly to provide more detail.

Minor point #19:

*Results in general – I appreciate the work you put into providing the vast amount of detail in the results. It is fairly lengthy and I am afraid you will begin to lose some of the readers because of its length (maybe I am wrong). Perhaps with better descriptors in your tables you can shift some of this information including p values and statistical significance for comparison in tables and reduce the text length.*

**Response:**

We thank Reviewer #1 for this comment. In an effort to reduce the length of the manuscript, some information was removed from the text when they were already presented in Tables 1 and 2. Furthermore, several other tables were added to lighten results text section.

Minor point #20:

*Line 433 can we separate this sentence up into 2? Also specify younger graduates are more likely when compared to who? We assume older graduates but please specify.*

**Response:**

As recommended, the sentence was separated, and we added that the younger graduates were compared to older graduates in Line 424-425 of revised manuscript.

Minor point #21:

*Line 439 – I get what you are saying but I do not think I would write that in an open access journal based on it being a big speculation.*

**Response:**

We thank Reviewer #1 for this comment. We understand that since this is a speculation it is not pertinent in an open access journal and the sentence was removed from revised manuscript (Line 428).

Minor point #22:

*Line 467 – I don’t agree with this statement. Yes, it does cause increased resistance but not if positive pressure ventilation is provided (by hand or machine). In addition the studies used to assess resistance are fairly old and used metal valves which were heavy. Our university does not use NRB circuits just because of the concern a student will accidently hit the quick flush.*

**Response:**

Even though it is possible to anaesthetise various patient sizes with only one type of anaesthetic system, it might be suboptimal to use Bain circuit in large patients or rebreathing system in small patients for the reasons mentioned in the manuscript. We understand Reviewer #1’s comment and we modified the paragraph starting Line 457-460 in revised manuscript to nuance our statements.

Minor point #23:

*Line 475 – I think simon et al 2017 on oligoanalgesia would be a good reference for this sentence.*

**Response:**

We thank Reviewer #1 for this comment. It is indeed a pertinent reference discussing the consequences of oligoanalgesia. Therefore, it was added to sentence in Line 465 of revised manuscript as Reference 21.

Minor point #24:

*Line 480-481 – I think you need to support this with a reference. Many areas routinely give them postoperatively so perhaps a brief explanation as to why this is inappropriate in Canada.’*

**Response:**

These sentences were modified to better express our thoughts in Line 469-470 of revised manuscript.

Minor point #25:

*Line 490 – lets avoid restating the results in the discussion please.*

**Response:**

The discussion was revised entirely to limit results repetition.

Minor point #26:

*Line 502 – inexpensive*

**Response:**

This orthographic error was corrected in Line 489 of revised manuscript.

Minor point #27:

*line 508 – to administer “fluid”? be more specific*

**Response:**

We thank Reviewer #1 for this comment. We specified “fluid therapy” instead of “fluid” in Line 495 of revised manuscript.

Minor point #28:

*line 516- thanks for pointing this out*

**Response:**

We thank Reviewer #1 for this comment. The authors felt indeed the need to bring this precision, so the reader doesn’t misinterpret the risks related to endotracheal intubation in cats for a recommendation against it.

Minor point #29:

*Line 521 – again too much results in the discussion. I understand there has to be some reference but in an already long manuscript I would try to reduce this when possible.*

**Response:**

To help decrease result repetition, several sentences were deleted from revised manuscript.

Minor point #30:

*Line 535 – who’s recommendations?*

**Response:**

It is American Animal Hospital Association’s recommendation. It is now specified in Line 518 of revised manuscript.

Minor point #30:

*line 602- please state a sig proportion of those surveyed… again I don’t think just over 100 vets is a sig portion of the population in Canada.*

**Response:**

The word “significant” was removed and it was specified that it’s the surveyed population that don’t respond to guidelines, as recommended in Line 584 of revised manuscript.

***Editor***

__________________________________________________________

*Dear Dr. Troncy,

Thank you for submitting your manuscript to PLOS ONE. After careful consideration, we feel that it has merit but does not fully meet PLOS ONE’s publication criteria as it currently stands. Therefore, we invite you to submit a revised version of the manuscript that addresses the points raised during the review process.*

**Response:**

We thank the Editor about his general comment concerning our manuscript. As recommended by the Reviewer, the modifications have been carried out and the essential revisions have been clarified in the answers addressed for each point. Thank you.
