# Supplementary material for: Management of veterinary anaesthesia and analgesia in small animals: A survey of English-speaking practitioners in Canada
Source: PLoS One. 2021 Sep 28;16(9):e0257448. doi: 10.1371/journal.pone.0257448 (PMC8478190; doi:10.1371/journal.pone.0257448)
Supplement: Attachment — Submitted filename: Response to reviewers PONE-D-21-05375_R1.docx [file pone.0257448.s007.docx]

**Response to the Reviewers’ comments on the manuscript**

**Title: Management of veterinary anaesthesia and analgesia in small animals: A survey of English-speaking practitioners in Canada**

**Ref:** PONE-D-21-05375R1

**Journal:** PLOS ONE

**Date:** 2021-08-30

______________________________________________________________________________

The authors would like to thank the Editorial Board and Reviewers for careful review of our manuscript and providing us with the comments to improve its quality. We have judiciously taken them into consideration in preparing our revision. The following detailed responses have been prepared to address the reviewer’s comments.

***Reviewer #1***

______________________________________________________________________________

**General comment:**

*Thank you so much for addressing all of my concerns. The manuscript is much approved. There are still some grammatical errors throughout that require the editors attention. I just had a few more minor comments.*

**Response:**

The authors thank the Reviewer for his/her general comment and rigorous revision about the present manuscript and the recognition that the main subject is of interest. We have taken care to address all comments to improve the quality and scientific thoroughness of our work.

Minor point #1:

*Line 20: Perhaps it is a common term in Canada (I will leave it up to the authors), but “English Canada” seems like an awkward term to me. Makes it sound like it’s a territory in Canada operated by the English.*

**Response:**

We agree with the Reviewer and have replaced “English Canada” for “English-speaking Canadian provinces”, which we feel is more accurate (Line 20).

Minor point #2:

*Line 21: SurveyMonkey® versus SurveyMonkey?*

**Response:** “®” symbol has been added accordingly (Line 21).

Minor point #3:

*Line 27: 126/? Respondents.*

**Response:** The number of invitations was added to specify denominator (Line 27). It was also added at Line 81 of revised manuscript.

Minor point #4:

*Line 75,87: SurveyMonkey® ?*

**Response:** “®” symbol has been added accordingly (Lines 75 and 87).

Minor point #5:

*Line 107: I guess I would consider these vets still working at the clinic, they just are not present at the time of the animal’s presentation.*

**Response:** The wording has been changed to be more specific (Line 107 in revised manuscript).

Minor point #6:

*Line 202: I think this is a confusing statement “(81% (38/47) use it more than 20% of times vs. 61% (30/49),”. What is confusing is “20% of times” do you mean “20% of the time”?*

**Response:** Here is how the question was presented in the survey:

“Question 29: Which of the following drugs are part of your premedication? (Please check all that apply). Please do not check unless certain to possess the selected drug. Frequency (%) of use in routine surgeries (0 %, 1-20 %, 21-40 %, 41-60, 61-80 %, 81-99 %, 100 %).”

Therefore, the sentence was changed with “20% of the time” Line 203.

Minor point #7:

*Line 204: same for here ”(22% (16/72) use it in more than 20% cases vs. 67% (6/9), P=0.023”. 20% of cases.*

**Response:** The wording has been changed accordingly, as it was done for Minor point #6 – See Lines 205 and 206.

Minor point #8:

*Line 225: This is why I asked about total participants “Respondents working in referral centres use ketamine-medetomidine (75% (6/8) vs. 29% 226 (16/56), P=0.016)”. I don’t understand how you found this to be statistically significant with only 8 individuals in 1 group. Population of 8 vs 56 doesn’t seem appropriate for comparison.*

**Response:** We decided to keep this statement in the result section because it was statistically significant. It was not discussed further in the discussion section because we agree with the reviewer that the clinical significance is weak, and would need to be confirmed. Such (statistically significant) result highlights the adequation of our statistical test to support non-identical sample size of compared (sub)groups. Please, don’t forget that this comparison focused on subpopulations of veterinarians (referral centres *vs*. GPs) for specific induction protocols. We thought such reported information to be attractive for description of differences in conditions of practice.

Minor point #9:

*Line 226: same as line 225.*

**Response:** See response to Minor point #8.

Minor point #10:

*Line 235: Again im not sure comparing such a small sample size to a large sample size is appropriate.*

**Response:** Similar to Minor points #8 and #9, this sentence was kept in the result section to include all statistically significant results. With only 13 respondents working in referral centers and 113 respondents working in general practices, this point applies to all comparisons between referral centers and general practices. We believe the type of practice was an important risk factor to study even with the low number of respondents in one category. The fact that such difference between practice conditions (referral centres *vs*. GPs) was not systematic on all analyzed components is justifying the presentation of statistically significant results as indicative of potentially real differences. Indeed, in a previous study, the type of practice had an influence on anaesthetic practice rules (Truchetti G, Otis C, Brisville AC, Beauchamp G, Pang D, Troncy E. Management of veterinary anaesthesia in small animals: A survey of current practice in Quebec. PLoS One. 2020;15(1):e0227204. doi: 10.1371/journal.pone.0227204). We added a comment in the discussion (Lines 580-584 in revised manuscript) to underline the limitation and stating that further studies should be done with a higher number of respondents to verify the findings.

***Editor***

__________________________________________________________

*Dear Dr. Troncy,

Thank you for submitting your manuscript to PLOS ONE. After careful consideration, we feel that it has merit but does not fully meet PLOS ONE’s publication criteria as it currently stands. Therefore, we invite you to submit a revised version of the manuscript that addresses the points raised during the review process.

The reviewer has still concerns regarding the statistical analysis in some areas (comparing small vs. larger groups). Please address these concerns in your discussion. Furthermore, the reviewer also noticed that there are many grammatical errors throughout your manuscript.*

**Response:**

We thank the Editor about his general comment concerning our manuscript. As recommended by the Reviewer, the modifications have been carried out and the essential revisions have been clarified in the answers addressed for each point. Thank you.
